# Investigation of Skeletal Muscle Indices Affecting Anaerobic Thresholds after Acute Myocardial Infarction

**DOI:** 10.1298/ptr.E10286

**Published:** 2024-07-09

**Authors:** Yuya UTSUMI, Koji TAKASE, Naoya MURAKAMI, Tokiko NAKAGAWA, Takuya OBAYASHI, Riyo OGURA, Shinobu HOSOKAWA

**Affiliations:** ^1^Department of Rehabilitation, Tokushima Red Cross Hospital, Japan; ^2^Department of Cardiology, Tokushima Red Cross Hospital, Japan

**Keywords:** Phase angle, Muscle quality, Anaerobic thresholds, Acute myocardial infarction

## Abstract

Objective: To investigate skeletal muscle indices influencing the anaerobic threshold (AT) measured by cardiopulmonary exercise testing (CPX). Methods: This study included 125 consecutive men (median age: 66.0 years) diagnosed with acute myocardial infarction who underwent CPX before discharge. Participants were categorized into two groups based on their AT: the AT-lowering and AT-maintaining groups, comprising those with AT <11 and ≥11 ml/min/kg, respectively. Skeletal muscle indices that influenced AT, strengths of such influences, and respective cutoff values were investigated using multiple logistic regression analysis, decision-tree analysis, and the random-forest method. Skeletal muscle indices included grip strength, knee extension strength, lower-limb skeletal muscle index, phase angle (PhA), lower-limb PhA, arm circumference (AC), and calf circumference. Results: Lower-limb PhA, AC, age, and body mass index (BMI) influenced AT (model X^2^ test: p <0.05; Hosmer-Lemeshow test: p = 0.98). Among the skeletal muscle indices, Gini impurity reduction was the highest in the lower-limb PhA. The cutoff values for AT were ≥4.0° for BMI <24 kg/m^2^ and ≥6.4° for BMI ≥24 kg/m^2^. Conclusion: Lower-limb PhA was the most influential skeletal muscle index affecting AT. PhA measured using body composition analyzers is useful to identify exercise-limiting factors and determine the effectiveness of exercise because it can be easily performed shortly.

## Introduction

Maximal oxygen uptake and anaerobic threshold (AT), measured using cardiopulmonary exercise testing (CPX), serve as key indicators of exercise tolerance and are considered prognostic factors in patients with cardiac disease^1^^,^^2)^. Cardiac, pulmonary, and skeletal muscle functions influence exercise tolerance^3–8)^. However, studies have reported that exercise rarely leads to significant improvements in left ventricular ejection fraction^9)^. Furthermore, several studies have suggested that cardiac function is not the primary limiting factor for exercise^10^^,^^11)^. Most studies reporting that exercise improved exercise tolerance in patients with heart disease also reported improvements in skeletal muscle indices such as muscle strength and calf circumference (CC)^12^^,^^13)^. Therefore, skeletal muscle function may be improved through exercise, which may play a crucial role in improving exercise tolerance.

Most of the reported associations between skeletal muscle function and exercise tolerance have been in elderly heart failure patients or heart failure patients with sarcopenia^5^^,^^7)^. However, many patients experiencing acute myocardial infarction (AMI) for the first time and younger patients with heart failure may exhibit exercise intolerance, as assessed by the CPX, even in the absence of decreased grip strength, lower extremity muscle strength, and skeletal muscle mass below the reference values. In particular, AMI is noted to occur at a younger age in men compared to women due to the influence of sex hormones, and in many cases, muscle strength and muscle mass are maintained^14)^. In such patients, identifying exercise-limiting factors and determining the effectiveness of exercise therapy may be difficult; it is hypothetically considered that conventional skeletal muscle indices may not sufficiently assess skeletal muscle function. Furthermore, CPX is currently performed in a limited number of facilities with a restricted number of patients because of the requirement for specialized equipment.

Identification of skeletal muscle indices that influence AT measured by CPX may contribute to the identification of exercise-limiting factors and the determination of the effectiveness of exercises. Therefore, this study aimed to identify skeletal muscle indices that influenced AT in patients with AMI.

## Methods

### Participants

**Fig. 1.**
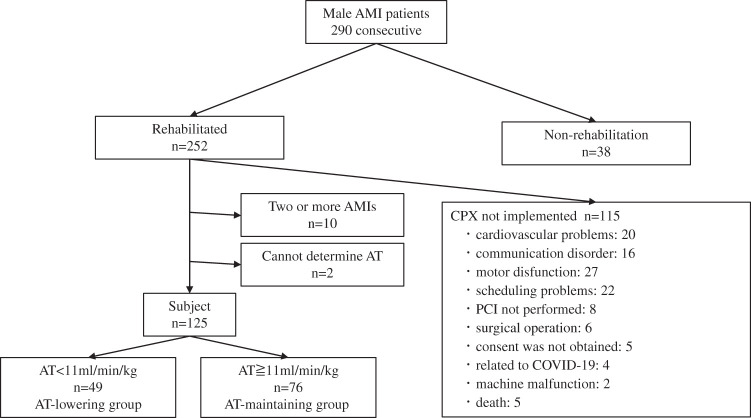
Flow diagram of the subject recruitment. AMI, acute myocardial infarction; AT, anaerobic threshold; CPX, cardiopulmonary exercise testing; PCI, percutaneous coronary intervention

Men diagnosed with AMI who presented to our hospital between July 2021 and July 2023 were included in the study (Fig. 1). Patients who did not have the opportunity to undergo rehabilitation, those who could not undergo CPX, and those with two or more AMIs were excluded. In previous studies on the relationship between AT and prognosis, an AT cutoff value of <11 kg/min/kg was demonstrated to be a good predictor of prognosis^15^^,^^16)^. Therefore, participants were categorized into two groups based on their AT: the AT-lowering and AT-maintaining groups, comprising those with AT <11 and ≥11 ml/min/kg, respectively.

Data used in this study originated from an ongoing prospective cohort study (Approval No: 758) that was approved by Ethics Committee of Tokushima Red Cross Hospital. Participants were informed of the study details and provided written consent according to an approved explanation and consent form.

### Assessments

The following skeletal muscle indices were measured approximately 3 days before discharge.

#### —Grip strength

A Smedley-type digital grip strength meter (Digital Grip-D; Takei Kiki, Niigata, Japan) was used for the measurements. The limb was kept in a standing position and measurements were performed once with the dominant hand.

#### —Isometric knee extension strength

Measurements were performed using a hand-held dynamometer (μTasF-100; Anima Corporation, Tokyo, Japan). The patient was seated on a bed with both upper limbs mildly abducted and the lower leg drooping. Isometric knee extension motion with maximal effort was performed once for approximately 5 seconds on the lower limb ipsilateral to the dominant hand. The value divided by the body weight was used due to the isometric knee extensor muscle strength.

#### —Arm and calf circumferences

Arm circumference (AC) and CC were measured at the middle of the upper arm and the maximum swelling of the lower leg, respectively, using a measuring tape.

#### —Skeletal muscle mass index and phase angle

Skeletal muscle mass index (SMI) was measured using a multi-frequency body composition analyzer (MC-780A-N; Tanita Corporation, Tokyo, Japan). Variables were measured according to a previously recommended method^17)^. Briefly, height was not self-reported but measured immediately before the SMI assessment. Measurements immediately after exercise and eating were avoided. In addition, the electrode contact area was disinfected with alcohol. For the measurements, the upper limbs were abducted 30° and separated from the trunk. SMI was calculated by dividing the amount of skeletal muscle in the four limbs by height squared according to the method recommended by the Asian Working Group for Sarcopenia^18)^. The lower-limb skeletal muscle index was calculated by dividing the amount of skeletal muscle in both lower limbs by height squared. In addition, the phase angle (PhA)—the ratio of resistance (resistance) between the cell’s outer and inner fluids and resistance (reactance) caused by the cell membrane at 50 kHz, expressed as an angle—was evaluated. The average PhAs of the left hemisphere and lower limb were used in subsequent analyses.

#### —CPX

For CPX measurements, an exercise stress electrocardiogram measurement device (STS-2100; Nihon Kohden, Tokyo, Japan), an ergometer for exercise stress tests (Strength Ergo 8 BK-ERG121; Fukuda Denshi, Tokyo, Japan), and an expiratory gas metabolism monitor (CPEX-1; Inter Reha, Tokyo, Japan) were used. The CPX protocol involved a 3-minute resting sitting position, followed by a 3-minute warm-up at 10 watts and a ramp load of 10 watts. The test concluded once AT evaluation was completed. AT was determined using the V-slope method or the widely used ventilatory equivalent method, as appropriate^19)^.

### Contents of rehabilitation

During hospitalization, patients underwent rehabilitation in accordance with the AMI rehabilitation program indicated by the attending physician. Our rehabilitation program was based on the program listed in the rehabilitation guidelines for patients with cardiovascular diseases^20)^. Most participants underwent either a 1-week or a 10-day program, which was performed once a day, 6 days a week. When the program could not be followed due to the implantation of a circulatory assist device or other reasons, exercise therapy was performed in consultation with the physician, as appropriate. Rehabilitation consisted of disease management guidance using pamphlets, basic movement practice, low-load resistance training, and gait practice, followed by aerobic exercise using a treadmill or bicycle ergometer in the rehabilitation room.

### Statistical analysis

Normally distributed continuous variables are expressed as mean ± standard deviation. while those with skewed distributions are expressed as median (interquartile range). Categorical variables were expressed as numbers (percentages).

To identify independent factors influencing AT (AT-lowering group vs. AT-maintaining group), a multiple logistic regression analysis was employed, in which the AT-maintaining group was coded as 1 and the AT-lowering group as 0. The independent variables included skeletal muscle indices, such as grip strength, knee extension strength, lower-limb SMI, PhA, lower-limb PhA, AC, and CC. Age and body mass index (BMI) were also included in the analysis. SMI was not included as an independent variable to account for multicollinearity.

In addition, a decision-tree analysis was used to calculate the optimal combination of variables and cutoff values that predicted an AT >11 ml/min/kg. Independent variables for this analysis were selected from the previous multiple logistic regression analysis. The existence of several nodes before reaching a leaf presents a risk of overlearning; therefore, it is desirable to reach a leaf with as few variables as possible.

Furthermore, variables that helped identify whether AT was >11 ml/min/kg were analyzed by Gini impurity reduction using a random-forest method. Independent variables for this analysis were selected from the previous multiple logistic regression analysis.

All analyses were performed using the Modified R commander version 4.0.2 for Mac OS. Statistical significance was set at p <0.05.

## Results

### Patient characteristics and physical functioning

A flowchart of the patient selection process is shown in Figure 1. Among the 290 consecutive men with AMI, 165 were excluded for not undergoing rehabilitation, CPX not being implemented, experiencing a second AMI, and the inability to determine the AT. Finally, 125 patients were included in the analyses. Of them, 49 and 76 participants were in the AT-lowering and AT-maintaining groups, respectively.

Tables 1 and 2 summarize the patient characteristics and their physical functioning, respectively. The median age of the participants was 66 (55–73) years, and there were no significant differences between the two groups (p = 0.35). The AT-lowering group exhibited a higher BMI, indicating a tendency toward obesity (p <0.05). In the AT-lowering group, participants more commonly underwent the 10-day program (p <0.05); however, no differences in Killip classification, intra-aortic balloon pumping (IABP) use, and peak creatine kinase (CK) were observed between the two groups, suggesting no difference in the severity of AMI. Although left ventricular ejection fraction was maintained in both groups, it was slightly lower in the AT-lowering group than in the AT-maintaining group (p <0.05).

**Table 1.** Patient’s characteristics

	All subjects n = 125	AT-maintaining group n = 76	AT-lowering group n =49	p
Basic information				
Age, years	66.0 (55−73.0)	64.0 (53.8−73.3)	58.0 (44.0−73.0)	0.35
BMI, kg/m^2^	24.1 (22.1−26.7)	23.1 (21.5−24.7)	25.4 (23.8−27.9)	<0.05
Emergency PCI Pre-emergency PCI	123 (97.6%) 3 (2.4%)	76 (100%) 0 (0%)	46 (93.9%) 3 (6.1%)	<0.05
Length of hospital, day	9 (7−11)	9 (7−11)	10 (8−12)	0.06
Killip’s classification	I: 102 (81.6%) II: 15 (12.0%) III: 1 (0.8%) IV: 7 (5.6%)	I: 66 (86.8%) II: 6 (7.9%) III: 1 (1.3%) IV: 3 (3.9)	I: 36 (73.5%) II: 9 (18.4%) III: 0 (0%) IV: 4 (8.2%)	0.17
IABP used	24 (19.2%)	15 (19.7%)	9 (18.4%)	0.85
Adaptation program	1 week: 80 (64.0%) 10 days: 41 (32.8%) Other: 4 (3.2%)	1 week: 57 (75.0%) 10 days: 17 (22.4%) Other: 2 (2.6%)	1 week: 23 (46.9%) 10 days: 24 (49.0%) Other: 2 (4.1%)	<0.05
Medical history				
Hypertension	87 (69.6%)	50 (65.8%)	37 (75.5%)	0.25
Diabetes mellitus	44 (35.2%)	24 (31.6%)	20 (40.8%)	0.29
Hyperlipidemia	78 (62.4%)	43 (56.6%)	35 (71.4%)	0.09
Currently smoking	53 (42.4%)	37 (48.7%)	16 (32.7%)	0.08
Various inspection results				
LVEF, %	58 (52−63)	59 (54−65)	56 (47−60)	<0.05
Peak CK, U/L	2334 (1315−3339)	2226 (1177−3137)	2334 (1391−3558)	0.46
STEMI	120 (96.0%)	74 (97.4%)	46 (93.9%)	0.33
Diseased branch	RCA: 52 (41.6%) LMT: 2 (1.6%) LAD: 52 (41.6%) LCX:18 (14.4%) HL:1 (0.8%)	RCA: 32 (42.1%) LMT: 0 (0%) LAD: 33 (43.4%) LCX: 11 (14.5%) HL: 0 (0%)	RCA: 20 (40.8%) LMT: 2 (4.1%) LAD: 19 (38.8%) LCX: 87 (14.3%) HL: 1 (2.0%)	0.31
Remnant branch	72 (57.5%)	39 (51.3%)	33 (67.3%)	0.13

Variables are expressed as mean ± standard deviation or median (interquartile range) or number of patients (%).

BMI, body mass index; PCI, percutaneous coronary angioplasty; IABP, intra-aortic balloon pumping; LVEF, left ventricular ejection fraction; peak CK, peak creatine kinase, STEMI, ST-elevation myocardial infarction; RCA, right coronary artery; LMT, left main coronary trunk artery; LAD, left anterior descending artery; LCX, left circumflex artery; HL, high lateral branch

**Table 2.** Patient’s physical function

	All subjects n = 125	AT-maintaining group n = 76	AT-lowering group n = 49	p
Skeletal muscle indices				
Assessment, hospital day	7 (5−8)	6 (5−7)	7 (6−10)	<0.05
Grip strength, kg	33.1 (28.8−38.8)	33.9 (29.2−38.1)	31.6 (27.6−41.2)	0.65
Knee extension per weight, %	41.1 ± 10.4	41.8 ± 9.0	40.0 ± 12.3	0.36
AC, cm	27.9 ± 2.8	27.4 ± 2.6	28.7 ± 2.9	<0.05
CC, cm	35.5 ± 3.2	34.9 ± 3.2	36.4 ± 2.8	<0.05
SMI, kg/m^2^	7.9 ± 0.9	7.9 ± 1.0	8.0 ± 0.9	0.38
Lower-limb SMI, kg/m^2^	6.1 ± 0.7	6.0 ± 0.8	6.2 ± 0.7	0.30
PhA,°	5.7 ± 0.8	5.8 ± 0.8	5.6 ± 0.8	0.21
Lower-limb PhA,°	5.1 ± 1.0	5.2 ± 1.0	4.9 ± 1.0	0.10
CPX				
Implemented, hospital day	7 (6−9)	7 (6−8)	8 (7−11)	<0.05
AT, ml/min/kg	11.8 (10.2−13.5)	12.9 (12.0−14.2)	10.0 (9.2−10.4)	<0.05

Variables are expressed as mean ± standard deviation or median (interquartile range).

AC, arm circumference; CC, calf circumference; SMI, skeletal muscle mass index; PhA, phase angle; CPX, cardiopulmonary exercise testing; AT, anaerobic threshold

The skeletal muscle index was measured on day 6 (5–7) in the AT-maintaining group and on day 7 (6–10) in the AT-lowering group (p <0.05). Both AC and CC were larger in the AT-lowering group, reflecting the tendency of participants to have obesity. CPX was performed on day 7 (6–8) in the AT-maintaining group and on day 8 (7–11) in the AT-lowering group (p <0.05).

### Multivariate analysis

The results of the multiple logistic regression analysis are presented in Table 3. Lower-limb PhA (odds ratio [OR]: 1.93, 95% confidence interval [CI]: 1.13−3.41, p <0.05) had a positive effect on AT. By contrast, age (OR: 0.95, 95% CI: 0.90−1.00, p <0.05), BMI (OR: 0.77, 95% CI: 0.63−0.93, p <0.05), and AC (OR: 0.79, 95% CI: 0.61−1.01, p = 0.06) had negative effects on AT.

**Table 3.** Multiple logistic regression analysis of AT and skeletal muscle indices

	Odds ratio	95% confidence interval	p
Lower-limb PhA,(°)	1.93	1.13–3.41	<0.05
Age, years	0.95	0.90–1.00	<0.05
BMI, kg/m^2^	0.77	0.63–0.93	<0.05
AC, cm	0.79	0.61–1.01	0.06

Model chi-square test p <0.05, Hosmer–Lemeshow test p = 0.98

PhA, phase angle; BMI, body mass index; AC, arm circumference

### Decision-tree analysis

**Fig. 2.**
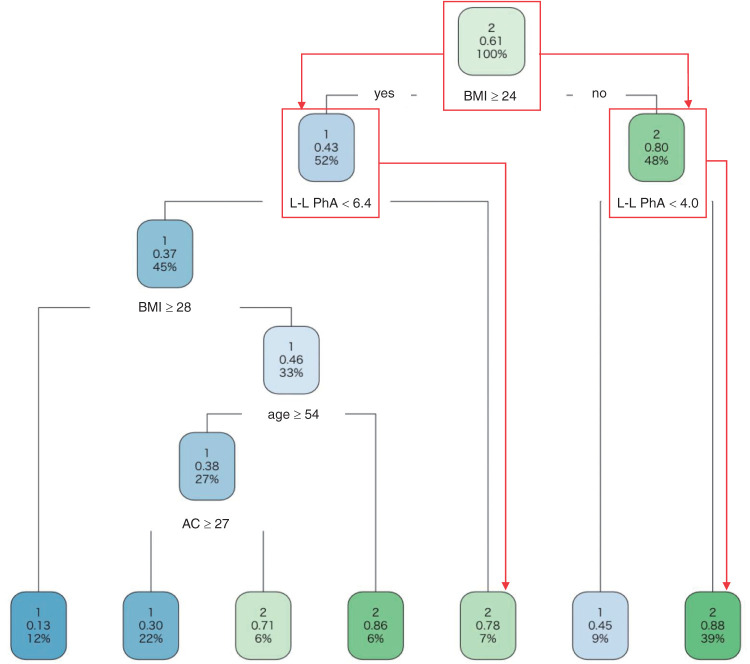
The result decision-tree analysis was used to calculate the optimal combination of variables and cutoff values that predicted an AT >11 ml/min/kg. The upper value in the node indicates that 2 represents AT ≥11 ml/min/kg, and 1 means <11 kg/min/kg. The value in the middle part of the node means the percentage that matches the criteria in the upper part of the node to the number of patients in the node. The value in the lower part of the node means the ratio of the number of patients matching the node’s criteria to the total number of patients. BMI, body mass index; AC, arm circumference, L-L PhA, lower-limb phase angle

The results of the decision-tree analysis are presented in Figure 2. The cutoff values of lower-limb PhA for predicting AT ≥11 mi/min/kg were ≥4.0° for those with BMI <24 kg/m^2^ and ≥6.4° for those with BMI ≥24 kg/m^2^.

### Random-forest analysis

**Fig. 3.**
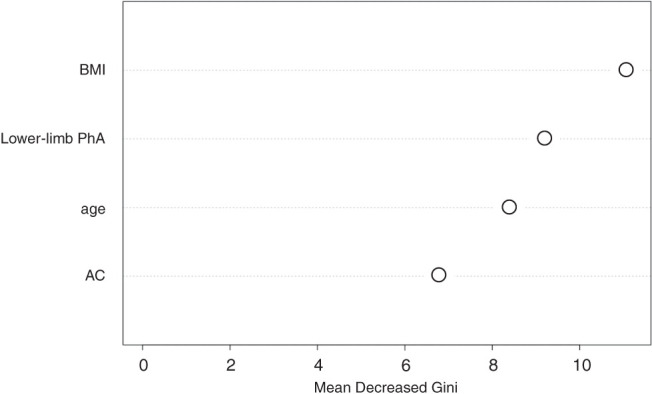
Result was analyzed by Gini impurity reduction using a random-forest method. A larger Gini impurity reduction indicates that the variable is more useful in identifying AT ≥11 ml/min/kg. BMI, body mass index; PhA, phase angle; AC, arm circumference

The results of the random-forest analysis are presented in Figure 3. Among the skeletal muscle indicators, lower-limb PhA was the most influential indicator of AT.

## Discussion

This study of patients with AMI investigated the skeletal muscle indices affecting AT, the respective cutoff values, and the strength of the effects. In the multivariate analysis, lower-limb PhA was observed to be the skeletal muscle index influencing AT; in the decision-tree analysis, it was also an important factor affecting AT. Furthermore, the random-forest method revealed that this factor strongly influenced AT compared with other skeletal muscle indices.

PhA is larger in men than in women and declines with age^21)^, and in healthy participants, it usually ranges between 5° and 7°^21)^. PhA is considered an indicator of skeletal muscle quality. Skeletal muscle quality has traditionally been determined using the amount of myosteatosis^22–24)^. Reportedly, myosteatosis assessed using ultrasound and computed tomography is associated with PhA^22–24)^, even after adjusting for age, sex, BMI, and SMI, which conceptually differs from skeletal muscle mass^23)^. In other words, regardless of the amount of skeletal muscle mass, a high amount of non-contractile tissue, such as that in myosteatosis, is considered to indicate a lower quality of skeletal muscle and vice versa. In addition, myosteatosis, assessed using computed tomography, has been reported to negatively affect walking speed and results of repetitive standing tests, independent of age and skeletal muscle area^24)^. This suggests that low muscle quality results in low PhA and has a negative effect on motor function. In CPX performed on a bicycle ergometer, lower-limb PhA was considered to affect AT because lower extremity muscles are primarily used in CPX.

PhA had a stronger effect on AT than knee extension muscle strength and grip strength, which are traditionally used skeletal muscle indices to reflect exercise capacity. There are two possible reasons for this. First, the AT measured by the CPX is physiologically different from conventionally used skeletal muscle indices in the context of athletic performance. AT is defined as the amount of oxygen consumption during exercise above which aerobic energy production is supplemented by anaerobic mechanisms^18)^. By contrast, maximal muscle strength measures, such as grip strength and knee extension strength, are instantaneous indices of skeletal muscle that are independent of aerobic metabolism. Another reason for the differences observed may be that the aforementioned studies used maximal oxygen uptake rather than AT as an index for exercise tolerance^5^^,^^6)^. Second, a mechanism of muscle weakness may be involved. In skeletal muscles, mitochondrial dysfunction is reported to precede a decline in muscle strength and mass^25)^. In other words, in the early stages of skeletal muscle dysfunction, there may be cases in which muscle strength is maintained but mitochondrial function is impaired. It was previously mentioned that PhA is an indicator of myosteatosis; additionally, studies in rats have suggested that mitochondrial dysfunction may cause myosteatosis^26)^. Although herein, the participants maintained muscle mass and skeletal muscle strength, it is possible that even in such cases, mitochondrial function was impaired, as reflected by PhA.

Decision-tree analysis revealed that the value of lower-limb PhA to predict AT ≥11 ml/min/kg differed between patients with BMI ≥24 kg/m^2^ and <24 kg/m^2^. In a previous study reporting the association between PhA and BMI by age, higher BMI (except for BMI≥35 kg/m^2^) was associated with greater PhA regardless of age^27)^. This is because reactance, one of the factors that determines PhA, is affected by the amount of cell membrane, and it has been reported that PhA increases with BMI^21)^. Therefore, our results corroborate previous studies showing that PhA values predicting AT vary with BMI.

Although PhA in healthy individuals ranges between 5° and 7°^21)^, PhA of diseased individuals varies among reports and diseases. Alves et al. reported the usefulness of PhA as a prognostic predictor in patients with heart failure, with 4.8° as the cutoff value^28)^. In addition, it has also been shown to be useful as a nutritional screening tool, with a reported cutoff value of 5°^29)^. Moreover, PhA is an independent predictor of 2-year survival in patients undergoing peritoneal dialysis, with a reported cutoff value of 6°^30)^. Thus, it is difficult to compare the PhA value to predict AT identified in this study with PhA values in other studies because the cutoff values of PhA vary depending on the target disease and outcomes.

The multiple logistic regression analysis showed that BMI and AC negatively affected AT (Table 3). This may be because AT comprises division by body weight; thus, it is likely to be underestimated in individuals with obesity. In addition, the BMI of the AT-lowering group was significantly higher than that of the AT-maintaining group (Table 1).

The results of this study indicate that PhA is an important predictor of exercise tolerance in individuals with preserved muscle strength and muscle mass, such as patients with a first myocardial infarction or young heart failure. In other words, even if skeletal muscle strength and muscle mass are maintained, a low PhA value may indicate low exercise tolerance due to skeletal muscle dysfunction. In addition, using PhA as an index to determine the effectiveness of exercise may enable accurate assessments of improvements in exercise tolerance. Because CPX requires specialized equipment, it cannot be performed in many facilities. It is also difficult to perform CPX frequently because it takes at least 30 minutes per session. Furthermore, there is a small possibility of adverse events^31)^. However, measuring PhA using body composition analyzers is easy, less time-consuming, and noninvasive. Therefore, PhA can be measured as a surrogate index of exercise tolerance, and AT≥11 ml/min/kg can be inferred if the cutoff values presented in this study are achieved. In other words, individuals with more than three METs at AT can safely perform most occupational, domestic, and leisure activities, which is useful for life guidance^32)^.

This study has some limitations. First, AT was used instead of peak oxygen consumption to measure exercise capacity. In general, peak oxygen consumption is often used as an indicator of exercise capacity^3–7)^. However, according to the guidelines for rehabilitation for cardiovascular disease, symptom-limited exercise testing after myocardial infarction can only be performed 14 days after onset^20)^. However, the length of hospital stay of the subjects in this study was approximately 9 days, making it difficult to perform CPX with symptom-limited exercise testing. In a clinical setting, multiple points sometimes imitate AT, making its determination difficult. Low muscle strength and gait speed have been reported as characteristics of patients in whom AT determination is difficult^33)^. However, this study included only a few such individuals, and AT could not be determined in only two participants (Fig. 1). Therefore, using AT as an index of exercise tolerance in individuals with preserved muscle strength and muscle mass, such as the participants in this study, is expected to have little influence on the results. Second, this study included a large proportion of participants with preserved muscle strength, and whether the same results would be obtained in patients with muscle weakness, such as sarcopenia, remains unclear. In cases of severe skeletal muscle impairment such as sarcopenia, exercise tolerance may be affected by traditional muscle strength and skeletal muscle mass rather than by PhA. Third, since this study included only men, whether the results are applicable to women remains unclear. In fact, the incidence is higher in older age groups than in men, suggesting that muscle strength and muscle mass may already be reduced in women^14)^. Therefore, conventionally used metrices such as muscle strength and muscle mass that are, rather than PhA, may have more influence on AT. In the future, the effect of PhA on women with AMI should be investigated. Fourth, we cannot rule out the possibility of overfitting in the results of the multivariate analysis in this study. It may be necessary to include more subjects and reexamine the issue.

Finally, this study investigated the association between AT and each skeletal muscle index in a cross-sectional manner. To our knowledge, there are no longitudinal studies regarding changes in PhA after AMI. Most studies reporting improvements in PhA with exercise used high-intensity resistance exercise in healthy individuals, and exercise programs adaptable to those with cardiac diseases are needed^34^^,^^35)^. In addition, it is necessary to examine whether improvements in PhA have a positive effect on exercise tolerance and patient prognosis.

## Conclusion

Herein, in men with AMI and preserved muscle strength and skeletal muscle mass, lower-limb PhA, which reflects muscle quality, was the skeletal muscle index with the most influence on AT. PhA measured using body composition analyzers is useful for identifying exercise-limiting factors and determining the effectiveness of exercise because it can be easily performed shortly.

## Funding

Not applicable.

## Conflicts of Interest

The authors declare no conflicts of interest.
